# Pulmonary alveolar proteinosis in Korea: analysis of prevalence and incidence via a nationwide population-based study

**DOI:** 10.1186/s12890-020-1074-5

**Published:** 2020-02-06

**Authors:** Hee-young Yoon, Ji Hyeon Kim, Ye-Jee Kim, Jin Woo Song

**Affiliations:** 1 Division of Pulmonary and Critical Care Medicine, Department of Internal Medicine, College of Medicine, Ewha Woman’s University, 25 Magokdong-ro 2-gil Gangseo-gu, Seoul, 07804 Republic of Korea; 2 Department of Pulmonary and Critical Care Medicine, Asan Medical Center, University of Ulsan College of Medicine, 88 Olympic-Ro 43-Gil, Songpa-Gu, Seoul, 05505 Republic of Korea; 3 Department of Clinical Epidemiology and Biostatistics, Asan Medical Center, University of Ulsan College of Medicine, 88 Olympic-Ro 43-Gil, Songpa-Gu, Seoul, 05505 Republic of Korea

**Keywords:** Epidemiology, Insurance claim review, Health care survey, National Health Programs, Rare diseases

## Abstract

**Background:**

Pulmonary alveolar proteinosis (PAP) is a very rare lung disease and its prevalence and incidence remain unclear. The prevalence and incidence of PAP were investigated by using nationwide claims data from the Korean Health Insurance Review and Assessment service.

**Methods:**

Data were extracted for adults who visited any secondary or tertiary medical institute between 2010 and 2016 with the PAP-related *Korean Classification of Disease, 7th edition* code J84.0 and the Rare Intractable Disease exempted calculation code V222. To robust case definition, a narrow case definition was made when all following factors were met: 1) more than two PAP-coded visits within 1 year of the first claim, and 2) more than one claim for both chest computed tomography and diagnostic procedures (bronchoscopy or surgical lung biopsy) within 90 days before or after the first claim.

**Results:**

A total of 182 patients (narrow, *n* = 82) with PAP-related codes were identified from 2010 to 2016 and 89 new patients (narrow, *n* = 66) visited medical institutes between 2012 and 2015. The prevalence of PAP was 4.44 (narrow: 2.27) per 10^6^ population, with a peak age of 60–69 years. The incidence of PAP was 0.56 (narrow: 0.41) per 10^6^ population at risk, with a peak age of 50–59 years. Among incident cases, the male-to-female ratio was 1.52 and about two-thirds had comorbidities, dyslipidaemia being the most common.

**Conclusions:**

The prevalence and incidence of PAP in Korea are low, similar to those in other countries; however, Korean patients with PAP are characterized by older diagnostic age and a lower male-to-female ratio.

## Background

Pulmonary alveolar proteinosis (PAP) is an extremely rare disease characterized by progressive accumulation of surfactant in the alveoli. PAP can affect all ages and sexes and shows diverse clinical courses, ranging from spontaneous resolution to respiratory insufficiency [1, 2]. Based on several case reports and series, the peak incidence of PAP is between 30 and 40 years and it predominantly occurs in men [2–10]. In previous studies, the prevalence of PAP was estimated to be 3.7–6.9 per 10^6^ population and incidence was estimated to be less than 1 person per 10^6^ population at risk [2–4, 11]; however, there are still limited data on the prevalence and incidence of PAP, especially in the Asian population.

In Asian countries including Korea, PAP was reported to be more prevalent in men, but the male-to-female ratio was relatively lower when compared with that of Western countries (1.3–2.1 [Asian] vs. 2.4–4.0 [Western]) [4, 5, 8, 10, 12, 13]. In addition, the age at diagnosis of Asian patients was usually higher (mean age: 47.5–52 vs. 34–49 years) [4, 5, 8, 10, 12, 13]. These results may reflect the genetic and environmental differences of Asian patients with PAP; however, these previous studies in Asia may have biased results since they included only selected patients who visited 10–15 referral medical institutes or those enrolled in the national PAP registry [4, 12, 13]. Therefore, in order to overcome these limitations, epidemiological studies based on entire populations are needed for rare diseases such as PAP.

More than 97% of the Korean population is covered by the National Health Insurance (NHI) system, which is a nationwide mandatory insurance scheme provided by the Korean government. Thus, claims data are appropriate for assessing the epidemiology of rare diseases in the whole population. The aim of this study was to estimate the prevalence and incidence of PAP in Korea by using the nationwide claims data.

## Materials

### Data source

From the Health Insurance and Review Agency (HIRA) database between January 2010 and December 2016, de-identified health claims data were obtained. This agency covers all health claims from the Korean NHI scheme and other available medical assistance programs (i.e., the Medical Assistance Program and Veterans Affairs Schemes) in South Korea. HIRA electronically collects the health claims data from medical institutions and stored data in the HIRA claims database. HIRA database includes all healthcare utilization information from inpatients and outpatients, including patient demographics, diagnosis, diagnostic procedures, and prescribed medication. The *seventh edition of the Korean Classification of Disease (KCD-7),* and the modification of *the 10th revision of the International Classification of Disease and Related Health Problems, (ICD-10)* were the sources for diagnostic codes (Additional file 1: Table S1).

Since 2010, PAP was added to the Rare Intractable Disease (RID) registration program, an NHI scheme initiated in 2006 with a current total of 167 rare intractable diseases. For patients to be registered into the RID program, a physician must confirm the diagnosis of PAP using the NHI criteria. The registered patients are then provided a co-payment reduction of 90% [14–17]. It is important to diagnose PAP correctly to meet all the NHI criteria, because medical institutions could not charge the NHI for medical expenses if a presumed diagnosis of PAP did not meet all the NHI criteria. Thus, all physicians involved strictly determined whether each patient qualified for registration in the RID system according to the standard NHI diagnostic criteria. Therefore, it is assumed that an accurate diagnosis must have been made at the time of RID registration. Also, previous studies have used the RID system to evaluate the prevalence and incidence of other rare diseases in Korea [14, 15, 17]. Following registration, both PAP-related *KCD-7* (J84.0) and RID codes (V222) were listed in PAP-related claims.

### Study population

All adult individuals (≥ 20 years) who visited the secondary or tertiary care medical institutions with the PAP-related KCD-7 code J84.0 as a diagnosis and the RID exempted calculation code V222 from 2010 to 2016 were included in the study. The NHI criteria required for PAP registration in the RID program are as follows: 1) compatible chest computed tomography findings with PAP and 2) bronchoalveolar lavage fluid or histopathologic findings compatible with PAP.

Since our study defined PAP cases structurally using claim data, narrow case definition was madden, to reduce possibility of overestimation or uncertainty of diagnosis. Following were the conditions used for narrow case definition; 1) at least two visits with a PAP-related code within a year of the first claim, and 2) claims of both chest computed tomography (CT) and diagnostic procedures (either bronchoscopy [BFS] or surgical lung biopsy) within 90 days before or after the first claim. The study was approved by the Institutional Review Board of Asan Medical Centre (2017–1190).

### Statistical analyses

For estimating prevalence, the patients identified with PAP during the study period were included in the prevalence estimates. Grounded on prevalent cases for 2010 and 2011, newly diagnosed patients from 2012 to 2016 were cumulatively added every year. The prevalence was calculated as follows: the number of total cases identified with PAP divided by the total population of South Korea for the year 2016. The Korean Statistical Information Service (http://kosis.kr) provided the 2016 population estimate. For the estimation of incidence, the date of the earliest claim of PAP was defined as the index date and the patient was regarded to be an incident case in that year. A clearance period was established by excluding cases identified in the first 2 years (2010–2011) to remove any potential pre-existing cases of PAP. The cases identified in 2016 were also excluded due to insufficient follow-up data. The annual incidence rate from 2012 to 2015 was calculated as follows: the number of newly identified cases in a corresponding year divided by the population at risk. The population at risk was calculated by removing the identified pre-existing cases of PAP from the mid-year population.

To compare the incidence rates over time, a standardized incidence for the Korean population in 2015 served as a reference, using direct standardization. The 95% confidence interval (CI) of prevalence and incidence rates was calculated using a Poisson distribution. For the newly diagnosed PAP cases, the age, sex, other accompanying diagnostic codes (comorbidities), and codes for diagnostic tests were analysed. The claims data on the diagnostic methods used were collected for 90 days before and after the first vist and the data on accompanying diseases were collected after the first visit. The SAS Enterprise Guide software (version 6.1, SAS Institute, Inc., Cary, NC, USA) was used to perform all statistical analyses.

## Results

### Prevalence

**Fig. 1:**
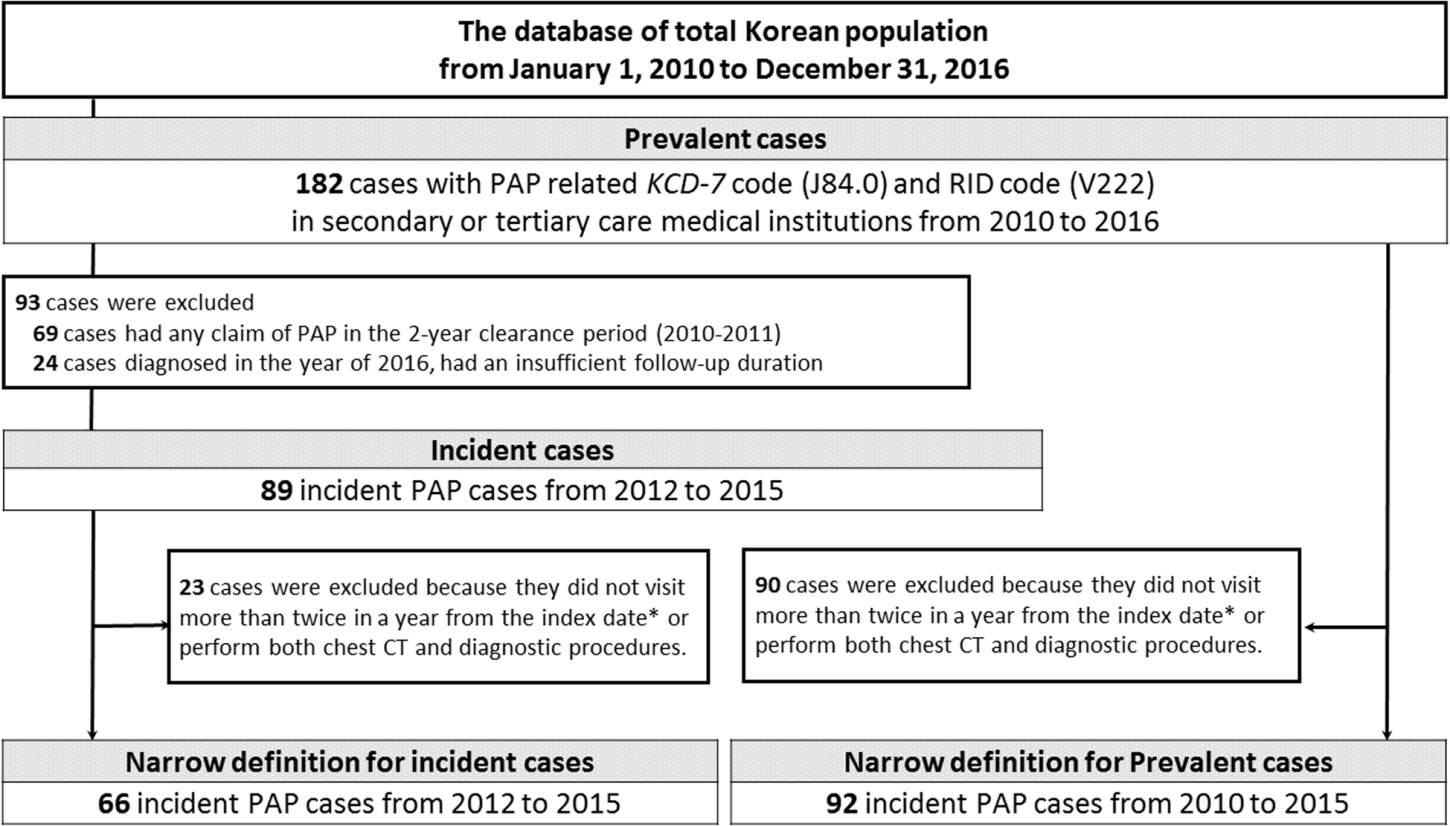
Identification of pulmonary alveolar proteinosis cases from the Health Insurance Review and Assessment Service database in Korea. PAP, pulmonary alveolar proteinosis; CT, computed tomography; KCD-7, Korean Classification of Disease, seventh edition; RID, The Rare Intractable Disease; *index date: the first claim of PAP

A total 182 prevalent cases (males: 119, females: 63) were identified from 2010 to 2016 (Fig. 1). The 7-year prevalence was 4.44 per 10^6^ people (95% CI: 3.82–5.14) in the whole population, 5.87 (95% CI: 4.86–7.03) in men and 3.05 (95% CI: 2.34–3.90) in women. Using the narrow case definition, 92 cases (males: 60, females: 32) were identified from 2010 to 2015 (Fig. 1) and the 7-year prevalence by the narrow definition was 2.27 (95% CI: 1.83–2.78) per 10^6^ people and that in men and women was 2.99 (95% CI: 2.28–3.85) and 1.59 (95% CI: 1.07–2.21) per 10^6^ people, respectively (Additional file 2: Table S2).

**Fig. 2:**
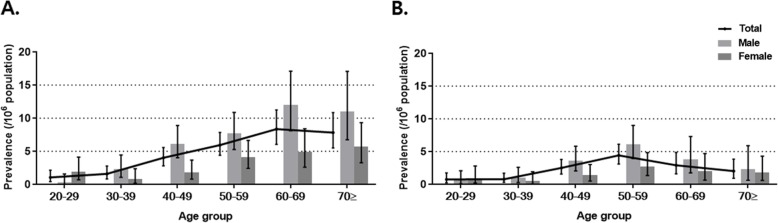
Age- and sex-stratified prevalence of pulmonary alveolar proteinosis **a** Prevalence from 2010 to 2016 **b** Prevalence of PAP from 2010 to 2015 based on the narrow definition. Data presented as the mean ± 95% confidence interval

The highest prevalence was observed in individuals aged 60–69 years (8.35 per 10^6^ people), followed by individuals aged > 70 (7.83 per 10^6^ people) (Additional file 2: Table S2). The peak prevalence in men was observed in individuals aged 60–69 years, whereas that in women was observed in individuals aged > 70 years. Using the narrow case definition, the peak prevalence age in the whole population, men, or women was 50–59 years, followed by 60–69 years (Fig. 2b).

### Incidence

Eighty-nine patients (males: 57, females: 32) were identified as incident cases between 2012 and 2015. The incidence rate was 0.56 (95% CI: 0.45–0.69) per 10^6^ population at risk in the whole population, and the incidence rates in men and women were 0.72 (95% CI: 0.55–0.94) and 0.40 (95% CI: 0.27–0.56) per 10^6^ population at risk, respectively (Additional file 3: Table S3). Using the narrow definition, the incidence rate was 0.41 (95% CI: 0.32–0.53) per 10^6^ population at risk in the whole population, 0.58 (95% CI: 0.43–0.78) in men, and 0.25 (95% CI: 0.15–0.38) in women.

**Fig. 3:**
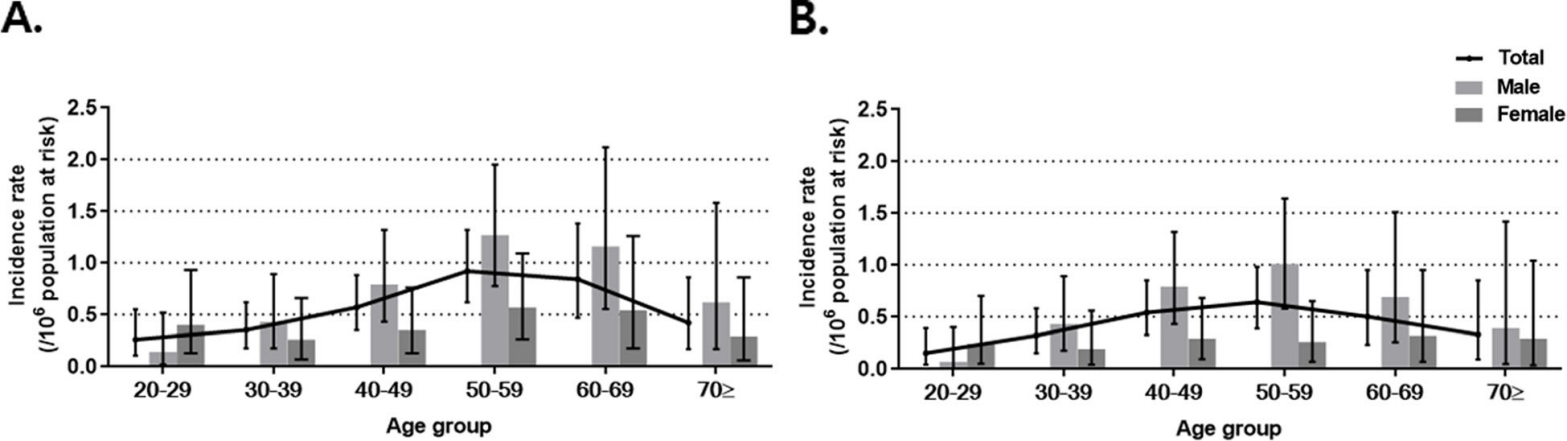
Age- and sex-stratified incidence rates of pulmonary alveolar proteinosis **a** Incidence rate from 2012 to 2015 **b** Incidence rate from 2012 to 2015 based on the narrow definition. Data presented as the mean ± 95% confidence interval

The age-specific incidence rate in the whole population and in both sexes was highest among 50–59 years (Fig. 3a; Additional file 3: Table S3). Using the narrow case definition, the peak incident rates in the total population and in men were highest in individuals aged 50–59 years, whereas that in women was highest in individuals aged 60–69 years (Fig. 3b).

### Changes in the annual incidence rates

**Fig. 4:**
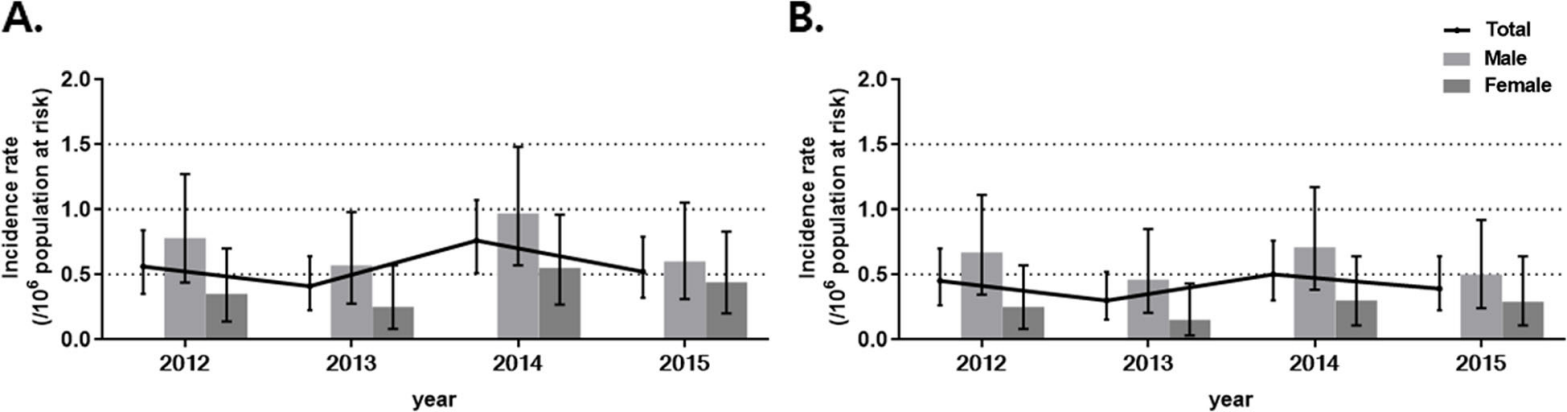
Age- and sex-standardized annual incidence rate of pulmonary alveolar proteinosis **a** Incidence rate from 2012 to 2015 **b** Incidence rate from 2012 to 2015 based on the narrow definition. Data presented as the mean ± 95% confidence interval

The age- and sex-standardized annual incidence rates were similar through the study period from 0.56 (95% CI: 0.35–0.84) per 10^6^ population at risk in 2012 to 0.52 (95% CI: 0.32–0.79) per 10^6^ population at risk in 2015 (Fig. 4a). Both sexes also showed similar findings. The standardized annual incidence rates in men were 0.78 (95% CI: 0.44–1.27) per 10^6^ population at risk in 2012 and 0.60 (95% CI: 0.31–1.05) per 10^6^ population at risk in 2015, and those in women were 0.35 (95% CI: 0.14–0.70] per 10^6^ population at risk in 2012 and 0.44 (95% CI: 0.20–0.83) per 10^6^ population at risk in 2015. The age- and sex-standardized annual incidence rates according to the narrow definition were also stable between 2012 and 2015 in all patients (from 0.45 [95% CI: 0.26–0.70] to 0.39 [95% CI: 0.23–0.64] per 10^6^ population at risk), in men (from 0.67 [95% CI: 0.35–1.11] to 0.46 [95% CI: 0.21–0.85] per 10^6^ population at risk), and in women (from 0.25 [95% CI: 0.08–0.57] to 0.29 [95% CI: 0.11–0.64] per 10^6^ population at risk) (Fig. 4b).

### Demographics and diagnostic methods in incident cases

The mean age of incident cases was 50.8 ± 13.4 years (men: 52.1 ± 11.8 years, women: 48.4 ± 15.8 years). The male-to-female ratio of incident cases was 1.82 and peaked at age 40–49 years. Of the incident cases, 92.1 and 70.8% had claims of the chest CT and BFS for 90 days before and after the first visit, respectively (Table 1). Surgical lung biopsy was performed in 34.8% of the incident cases.

**Table 1:** Methods used for diagnosing incident pulmonary alveolar proteinosis cases

Diagnostic methods	N (%)
Chest computed tomography	82 (92.1)
Bronchoscopy	63 (70.8)
Bronchoalveolar lavage	56 (62.9)
Surgical lung biopsy	31 (34.8)

Data are presented as total number (%)

N, total number

### Comorbidities of incident cases

The most common accompanying diagnostic code encountered in the incident cases was dyslipidaemia (E78, 60.7%), followed by diabetes mellitus (E10–14, 40.5%), and hypertension (I10–15, 40.5%) (Table 2). Among respiratory comorbidities, chronic obstructive pulmonary disease (COPD, J44, 23.6%) was the most common, followed by tuberculosis (A15–19, 5.6%), and non-tuberculous mycobacteria (A31.9, 1.1%). (Table 2).

**Table 2:** Accompanying diagnostic codes in incident pulmonary alveolar proteinosis cases after registration

	Total	Male	Female
Dyslipidaemia (E78)	54 (60.7)	38 (66.7)	16 (50.0)
Diabetes mellitus (E10–14)	36 (40.5)	26 (45.6)	10 (31.3)
Hypertension (I10–15)	36 (40.5)	24 (42.1)	12 (37.5)
Chronic obstructive pulmonary disease (J44)	21 (23.6)	17 (29.8)	4 (12.5)
Ischaemic heart disease (I20–25)	11 (12.4)	9 (15.8)	2 (6.3)
Malignancy (C00–97)	9 (10.1)	4 (7.0)	5 (15.6)
Tuberculosis (A15–19)	5 (5.6)	4 (7.0)	1 (3.1)
Non-tuberculous mycobacteria (A31.9)	1 (1.1)	1 (1.8)	0 (0.0)
Arrhythmia (I47–49)	4 (4.5)	3 (5.3)	1 (3.1)
Renal failure (N17–19)	3 (3.4)	2 (3.5)	1 (3.1)
Asbestosis (J61)	1 (1.1)	1 (1.8)	0 (0.0)

Data are presented as number (%)

## Discussion

This study is the first population-based study assessing the prevalence and incidence of PAP in Korea using data from a nationwide medical claims database. The prevalence and incidence of PAP in Korea were low, similar to those of other countries, but characterized by older diagnostic age and lower male to female ratio when compared to other countries. The annual incidence rate was steady during the study period and middle-aged men showed the highest risk.

Our results showed a low prevalence of PAP in Korea (4.44 per 10^6^ people from 2010 to 2016), similar to that in Japan [4, 11]. Inoue Y. et al., using national cohort data from 9 primary clinical research centres in Japan, reported that the prevalence of autoimmune PAP was 6.2 per 10^6^ population between 1999 and 2006 [4]. The results from Western countries are also compatible with our findings. Ben-Dov I. et al., based on the results from a nationwide survey, showed that the prevalence of PAP in Israel from 1976 to 1998 was 3.7 per 10^6^ population [3]. In the study using the health insurance claims data covering approximately 30 million people in the United States, the prevalence of PAP in the United States was reported as 6.9 per 10^6^ population between 2008 and 2012 [11]. In addition, the incidence rate of PAP in our study (0.56 per 10^6^ population at risk) was similar to that in other countries (0.49 [Japan], 0.36 [Israel]) [3, 4], suggesting that the racial differences of the epidemiology of PAP are not large. However, the demographic features of incident cases in our study showed different results; the mean diagnostic age of this study was higher (50.8 vs. 34–49 years), and the male to female ratio was lower (1.8 vs. 2.4–4.1) than the results of Western countries [5, 10, 18, 19]. The study in Japan, however, showed similar findings to our results; the mean diagnostic age was 51 years and the male to female ratio was 2.1 [4]. These results suggest that ethnicity may have an impact on the demographic features of PAP.

The age at diagnosis of PAP in our study was similar to that in previous Korean studies [12, 13]. Byun et al., using a cohort of 38 Korean patients who were diagnosed with PAP in 10 secondary and tertiary referral hospitals between 1993 and 2007, showed that the median diagnostic age of PAP was 52 years [12]. Hwang et al., using a cohort of 78 PAP patients diagnosed between 1993 and 2014, also reported similar results (median: 47.5 years, interquartile range: 42.5–59 years) [13]. However, another study, using a cohort with 12 PAP patients diagnosed between 1987 and 1998, showed a younger diagnostic age (median: 43.5 years, interquartile range: 33.0–51.5) [20] when compared with the previously mentioned recent studies [12, 13]. These findings might be attributed to the increase of the aging population in Korea; the proportion of the elderly (≥ 65 years) increased from 7.2% in 2000 to 13.8% in 2017 (Statistics Korea, http://kostat.go.kr/). In addition, since 2007, the Korean government has been offering regular health check-ups, including a chest x-ray every 2 years to all Koreans > 40 years of age; therefore, PAP patients > 40 years of age are more readily detected. This could explain the late diagnostic age of PAP observed in our current study. Recent prevalence of PAP in United States between 2008 and 2012 peaked at over 75 years followed by the aged 65–74 years also supports the global aging of PAP patients [11].

In this study, the patients diagnosed with PAP commonly had accompanied dyslipidaemia (60.7%) and diabetes (40.5%). In the Korean population, the prevalence of hypercholesterinaemia, hypertriglyceridemia, and diabetes were reported to be 19.9, 17.2, and 11.3%, respectively in 2016, suggesting an increased prevalence of metabolic disorders in Korean patients with PAP when compared with the general population. Previous studies support our findings; in a study involving non-obese and non-diabetic PAP patients (*n* = 33), Tian et al., demonstrated increased triglyceride levels (median: 192.0 mg/dl vs. 119.6 mg/dl, *p* < 0.05) and reduced high-density lipoprotein cholesterol levels (42.5 mg/dl vs. 51.4 mg/dl, *p* < 0.01) when compared with blood pressure- matched healthy controls (*n* = 157) [21]. This finding might be linked to the cholesterol-lowering effect of GM-CSF by promoting macrophage functions in lipid metabolism [22]. GM-CSF (*Csf2*) knockout (*Csf2*−/−*)* and Csf2rb gene-deficient (*Csf2r*b−/−) mice showed surfactant-containing macrophages with excessive accumulation of cholesterol ester-rich lipid-droplets [23]. McCarthy et al., in a study using the claims data, also reported that PAP patients had a higher rate of diabetes (26.9% vs. 13.4%) than the US general population [11]. The insulin resistance observed in these diabetics could be attributed to excessive lipid metabolites or cytokines inhibiting insulin signal [24, 25].

In our PAP patients, mycobacterial infection was accompanied by about 5%. Several studies reported the association between tuberculosis (TB) and PAP [26–29]. Zhang et al. reported that TB infection was identified in 44% of secondary PAP patients (*n* = 9) [26]. Some coexisting cases of PAP and TB have also been reported [27–29]. In the experimental study using in vivo model, GM-CSF production inhibited mycobacterial growth by mediating invariant natural killer T cells [30] and in other study, GM-CSF deficient mice fail to control mycobacterial infection due to the reduced lymphocytes recruitments and T helper cell type 1 response [31]. No specific associations between PAPs and other accompanying diseases (*COPD, hypertension, ischemic heart disea*se)) have been reported, however, clinical characteristics of our incidence cases (the predominance of middle aged-men) may contribute to the coexistence of both diseases.

This study had an increased proportion of subjects who underwent diagnostic bronchoscopy (70.8% vs. 41.7–83.3%) when compared with previous studies [12, 13, 20]; however, the number of subjects who underwent surgical lung biopsy decreased (34.8% vs. 42.0–58.3%). In a review study based on patients with PAP between 1958 and 1988, surgical lung biopsy (71%) was the main diagnostic tool, while the proportion of bronchoscopy (10%) for diagnosis was low [2]. However, compared with this earlier study [2], recent studies showed relatively high rates of bronchoscopy (56–97% [recent] vs. 10% [earlier]) and low rates of surgical lung biopsy (6–34% vs. 71%) for PAP diagnosis [4, 18, 19]. These results collectively suggest that non-invasive tests are used more actively for the diagnosis of PAP due to increased experience [32].

Several limitations must be considered. Firstly, PAP cases were defined by using diagnostic codes from a national insurance claims database. Since this database did not provide information for personal identification to researchers due to the Korean Personal Information Protection Act, validation through medical chart review was not possible for the diagnosis of PAP cases. Consequently, this lack of validation may have led to an overestimation of the prevalence and incidence rates. To compensate for this shortcoming, the RID registration system, which demanded the subject to satisfy the universal diagnostic criteria for registration and to be reviewed by the medical institutions before submission to the NHI system, wss used. To calculate more accurate the incidence and prevalence of PAP, we also used the narrow definition of PAP. Secondly, our study only included the PAP patients who visited the secondary or tertiary medical institutes. This inclusion criterion might have led to selection bias in that only severe PAP cases were included. However, due to its rarity, it is unlikely that a diagnosis of PAP was made at primary medical institutions. Even if such diagnoses were made, their accuracy would have been a cause of concern. Thus, it was appropriate to exclude patients diagnosed at these primary medical institutions. Despite these limitations, our study yielded unbiased results from the entire population.

## Conclusion

Our results suggest that the epidemiology of PAP in Korea estimated using the national insurance claims data is similar to that in other countries; however, the age at diagnosis is higher and the male-to-female ratio is lower when compared with those in Western countries.

## Supplementary information

**Additional file 1: Table S1.** Matching diagnostic codes between the KCD-7 and ICD-10 classifications.

**Additional file 2: Table S2.** Number of patients with pulmonary alveolar proteinosis and prevalence (per 10^6^ population) in Korea from 2010 to 2016.

**Additional file 3: Table S3.** Number of patients with pulmonary alveolar proteinosis and incidence rate (per 10^6^ population at risk) in Korea from 2012 to 2015.

## Data Availability

Any data generated and/or analysed during the current study are available from the corresponding author on reasonable request.

## Abbreviations

BFS: Bronchoscopy
CI: Confidence interval
CT: Computed tomography
HIRA: Health Insurance and Review Agency
KCD: Korean Classification of Disease
NHI: National Health Insurance
PAP: Pulmonary alveolar proteinosis
RID: Rare Intractable Disease+
