# Supplementary material for: Pulmonary alveolar proteinosis in Korea: analysis of prevalence and incidence via a nationwide population-based study
Source: BMC Pulm Med. 2020 Feb 6;20:34. doi: 10.1186/s12890-020-1074-5 (PMC7006195; doi:10.1186/s12890-020-1074-5)
Supplement: Supplementary file 1 — Additional file 1: Table S1. Matching diagnostic codes between the KCD-7 and ICD-10 classifications. [file 12890_2020_1074_MOESM1_ESM.docx]

**Pulmonary alveolar proteinosis in Korea: Analysis of prevalence and incidence via a nationwide population-based study**

Hee-young Yoon, Ji Hyeon Kim, Ye-Jee Kim, Jin Woo Song

**Table S1.** Matching diagnostic codes between the *KCD-7* and *ICD-10* classifications

| KCD-7 | ICD-10 |
| --- | --- |
| A15-19 Tuberculosis | A15-19 Tuberculosis |
| A 31.9 Mycobacterial infection, unspecified | A 31.9 Mycobacterial infection, unspecified |
| Atypical mycobacteriosis, familial, X-linked | Atypical mycobacteriosis, familial, X-linked |
| Atypical mycobacterial infection NOS | Atypical mycobacterial infection NOS |
| Mycobacteriosis NOS | Mycobacteriosis NOS |
| C00-C97 Malignant neoplasms | C00-C97 Malignant neoplasms |
| E10-E14 Diabetes mellitus | E10-E14 Diabetes mellitus |
| E78 Disorders of lipoprotein metabolism and other lipidaemias | E78 Disorders of lipoprotein metabolism and other lipidaemias |
| I10-I15 Hypertensive diseases | I10-I15 Hypertensive diseases |
| I20-I25 Ischaemic heart diseases | I20-I25 Ischaemic heart diseases |
| I47 Paroxysmal tachycardia | I47 Paroxysmal tachycardia |
| I48 Atrial fibrillation and flutter | I48 Atrial fibrillation and flutter |
| I49 Other cardiac arrhythmias | I49 Other cardiac arrhythmias |
| J44 Other chronic obstructive pulmonary disease | J44 Other chronic obstructive pulmonary disease |
| J61 Pneumoconiosis due to asbestos and other mineral fibres | J61 Pneumoconiosis due to asbestos and other mineral fibres |
| J84.0 Alveolar and parietoalveolar conditions | J84.0 Alveolar and parietoalveolar conditions |
| Alveolar proteinosis | Alveolar proteinosis |
| Pulmonary alveolar microlithiasis | Pulmonary alveolar microlithiasis |
| N17-N19 Renal failure | N17-N19 Renal failure |

*KCD-7*, *Korean Classification of Disease, 7^th^ revision*; *ICD-10*, *International Classification of Disease, 10^th^ revision*.
