# Supplementary material for: Pulmonary alveolar proteinosis in Korea: analysis of prevalence and incidence via a nationwide population-based study
Source: BMC Pulm Med. 2020 Feb 6;20:34. doi: 10.1186/s12890-020-1074-5 (PMC7006195; doi:10.1186/s12890-020-1074-5)
Supplement: Supplementary file 2 — Additional file 2: Table S2. Number of patients with pulmonary alveolar proteinosis and prevalence (per 106 population) in Korea from 2010 to 2016. [file 12890_2020_1074_MOESM2_ESM.docx]

**Pulmonary alveolar proteinosis in Korea: Analysis of prevalence and incidence via a nationwide population-based study**

Hee-young Yoon, Ji Hyeon Kim, Ye-Jee Kim, Jin Woo Song

**Table S2.** Number of patients with pulmonary alveolar proteinosis and prevalence (per 10^6^ population) in Korea from 2010–2016

| Age group | Total | | | | Male | | | | Female | | | | Male-to-female ratio |
| --- | --- | --- | --- | --- | --- | --- | --- | --- | --- | --- | --- | --- | --- |
|  | N | Total population | P | 95% CI | N (%) | Total population | P | 95% CI | N (%) | Total population | P | 95% CI |  |
| 20–29 years | 7 | 6,695,952 | 1.05 | 0.42-2.15 | 1 | 3,526,732 | 0.28 | 0.01-1.58 | 6 | 3,169,220 | 1.89 | 0.69-4.12 | 0.15 |
| 30–39 years | 12 | 7,538,707 | 1.59 | 0.82-2.78 | 9 | 3,850,486 | 2.34 | 1.07-4.44 | 3 | 3,688,221 | 0.81 | 0.17-2.38 | 2.87 |
| 40–49 years | 35 | 8,716,227 | 4.02 | 2.80-5.58 | 27 | 4,418,589 | 6.11 | 4.03-8.89 | 8 | 4,297,638 | 1.86 | 0.80-3.67 | 3.28 |
| 50–59 years | 49 | 8,247,924 | 5.94 | 4.40-7.85 | 32 | 4,155,589 | 7.70 | 5.27-10.87 | 17 | 4,092,335 | 4.15 | 2.42-6.65 | 1.85 |
| 60–69 years | 43 | 5,151,264 | 8.35 | 6.04-11.24 | 30 | 2,502,779 | 11.99 | 8.09-17.11 | 13 | 2,648,485 | 4.91 | 2.61-8.39 | 2.44 |
| ≥ 70 years | 36 | 4,595,328 | 7.83 | 5.49-10.85 | 20 | 1,809,383 | 11.05 | 6.75-17.07 | 16 | 2,785,945 | 5.74 | 3.28-9.33 | 1.92 |
| Total | 182 | 40,945,401 | 4.44 | 3.82-5.14 | 119 | 20,263,558 | 5.87 | 4.86-7.03 | 63 | 20,681,844 | 3.05 | 2.34-3.90 | 1.93 |

N, number. P, Prevalence. CI, confidence interval.
