# Supplementary material for: Pulmonary alveolar proteinosis in Korea: analysis of prevalence and incidence via a nationwide population-based study
Source: BMC Pulm Med. 2020 Feb 6;20:34. doi: 10.1186/s12890-020-1074-5 (PMC7006195; doi:10.1186/s12890-020-1074-5)
Supplement: Supplementary file 3 — Additional file 3: Table S3. Number of patients with pulmonary alveolar proteinosis and incidence rate (per 106 population at risk) in Korea from 2012 to 2015. [file 12890_2020_1074_MOESM3_ESM.docx]

**Pulmonary alveolar proteinosis in Korea: Analysis of prevalence and incidence via a nationwide population-based study**

Hee-young Yoon, Ji Hyeon Kim, Ye-Jee Kim, Jin Woo Song

**Table S3.** Number of patients with pulmonary alveolar proteinosis and incidence rate (per 10^6^ population at risk) in Korea from 2012–2015

| Age group | Total | | | | Male | | | | Female | | | | Male-to-female ratio |
| --- | --- | --- | --- | --- | --- | --- | --- | --- | --- | --- | --- | --- | --- |
|  | N | Population at risk* | I | 95% CI | N (%) | Population at risk* | I | 95% CI | N (%) | Population at risk* | I | 95% CI |  |
| 20–29 years | 7 | 26,443,780 | 0.26 | 0.11-0.55 | 2 | 13,848,497 | 0.14 | 0.02-0.52 | 5 | 12,595,283 | 0.40 | 0.13-0.93 | 0.36 |
| 30–39 years | 11 | 31,676,928 | 0.35 | 0.17-0.62 | 7 | 16,146,893 | 0.43 | 0.17-0.89 | 4 | 15,530,035 | 0.26 | 0.07-0.66 | 1.68 |
| 40–49 years | 20 | 35,064,091 | 0.57 | 0.35-0.88 | 14 | 17,824,374 | 0.79 | 0.43-1.32 | 6 | 17,239,717 | 0.35 | 0.13-0.76 | 2.26 |
| 50–59 years | 29 | 31,472,892 | 0.92 | 0.62-1.32 | 20 | 15,804,005 | 1.27 | 0.77-1.95 | 9 | 15,668,888 | 0.57 | 0.26-1.09 | 2.20 |
| 60–69 years | 15 | 17,915,601 | 0.84 | 0.47-1.38 | 10 | 8,657,148 | 1.16 | 0.55-2.12 | 5 | 9,258,453 | 0.54 | 0.18-1.26 | 2.14 |
| ≥ 70 years | 7 | 16,724,121 | 0.42 | 0.17-0.86 | 4 | 6,471,218 | 0.62 | 0.17-1.58 | 3 | 10,252,903 | 0.29 | 0.06-0.86 | 2.11 |
| Total | 89 | 159,297,412 | 0.56 | 0.11-0.69 | 57 | 78,752,134 | 0.72 | 0.55-0.94 | 32 | 80,545,278 | 0.40 | 0.27-0.56 | 1.82 |

N, number. I, Incidence rate. CI, confidence interval.

*population at risk was defined by removing prevalent pulmonary alveolar proteinosis cases from the mid-year population number
